# Supplementary material for: Enhancement of Mycelial Growth and Antifungal Activity by Combining Fermentation Optimization and Genetic Engineering in Streptomyces pratensis S10
Source: Microorganisms. 2025 Aug 20;13(8):1943. doi: 10.3390/microorganisms13081943 (PMC12388169; doi:10.3390/microorganisms13081943)
Supplement: Supplementary file 1 [file microorganisms-13-01943-s001.zip › microorganisms-3782251-supplementary.pdf]

microorganisms

Supplementary Materials

1. Antifungal Activity Assay

Fermentation culture was centrifuged at 5000 rpm and 4℃ for 10 min to collect the pellet. The precipitate was resuspended in three volumes of methanol (MeOH) and subjected to ultrasonication for 10 min. The resulting mixture was filtered through filter paper, and the MeOH extract was condensed using a rotary vacuum evaporator to obtain dried residues. The extract was redissolved in MeOH to prepare a stock solution at a final concentration of 50 mg/mL. The antifungal activity was evaluated as previously described [1]. Briefly, the MeOH extract was incorporated into 20 mL of sterile PDA medium at a final concentration of 50 μg/mL, with an equivalent volume of MeOH serving as a negative control. A 6-mm fresh mycelial plug was taken from the edge of 3-day-old fungal colony, placed on the center of each plate. After incubation at 28℃ for 5 days, the colony diameter was measured. Each treatment was performed in triplicate, and the experiment was repeated three times independently. The inhibition rate was calculated by the following:

Inhibition rate (%) = (the colony diameter of control — the colony diameter of treatment)/ (the colony diameter of control) × 100

2. Single-factor concentration screening test

Systematic optimization of key medium components was performed via a single-variable approach. The basal medium was supplemented with varying concentrations of each components as follows: corn flour (0, 5, 10, 20, 40 and 80 g/L), KCl (0, 0.125, 0.25, 0.5, 1 and 2 g/L), yeast extract (0, 5, 10, 20, 40 and 80 g/L), $NaNO_3$ (0, 0.25, 0.5, 1, 2 and 4 g/L), $ZnSO_4{\cdot}7H_2O$ (0, 0.0025, 0.005, 0.010, 0.020 and 0.040 g/L), $CaCO_3$ (0, 0.125, 0.25, 0.5, 1.0 and 2.0 g/L) and $MnCl_2{\cdot}4H_2O$ (0, 0.0025, 0.005, 0.010, 0.020 and 0.040 g/L). The mycelium dry weight and antifungal activity were assessed to determine the optimal concentration for each factor.

Table S1 The level and code of variables using the PBD

Name	Factors	Code Value（g/L）	
		-1	+1
A	Corn flour	20	25
B	Yeast extract	5	6.25
C	$NaNO_3$	1	1.25
D	$CaCO_3$	0.5	0.625
E	KCl	2	2.5
F	$ZnSO_4{\cdot}7H_2O$	0.02	0.025
G	$MnCl_2{\cdot}4H_2O$	0.02	0.025

Table S2 Level and code of variables chosen for BBD

Name	Factors	Code Value （g/L）		
		-1	0	1
A	Corn flour	27	29.5	30.5
B	Yeast extract	8.5	10.0	10.5
C	$ZnSO_4{\cdot}7H_2O$	0.0195	0.0295	0.0395

Table S3 Primers used in this study

Gene	Forward primer sequence	Reverse primer sequence
Arm	ACGACGGCCAGTGCCAAGCTGCG-CATTCCGAGAAGAAGGA	TTTCCACGGTGTGCGTCCGCCCGCCGATTTCTG-TAGTGCT
Brm	AAATTGTCACAAC-GCCGCGGTCAACTCCCTCTCCCTCCAA	CTATGACATGATTACGAATTT-GATGTCGCTGGTCGAAGTC
Gm	AGCACTACAGAAATCGGCGGGCGGAC-GCACACCGTGGAAA	TTGGAGGGAGAGGGAGTTGACCGCGGCGTT-GTGACAATTT
tet	CGAGGATCGTGGACCAGTG	GTCGACGAACTGGTAGTTGACG
Com	ACAGCTACGCCAAGGACGAC	CGCGTCCTAATCTAAGGGCAG
Apra	GAGTGCAATGTCGTGCAATACGA	GCATTCTTCGCATCCCGCCT

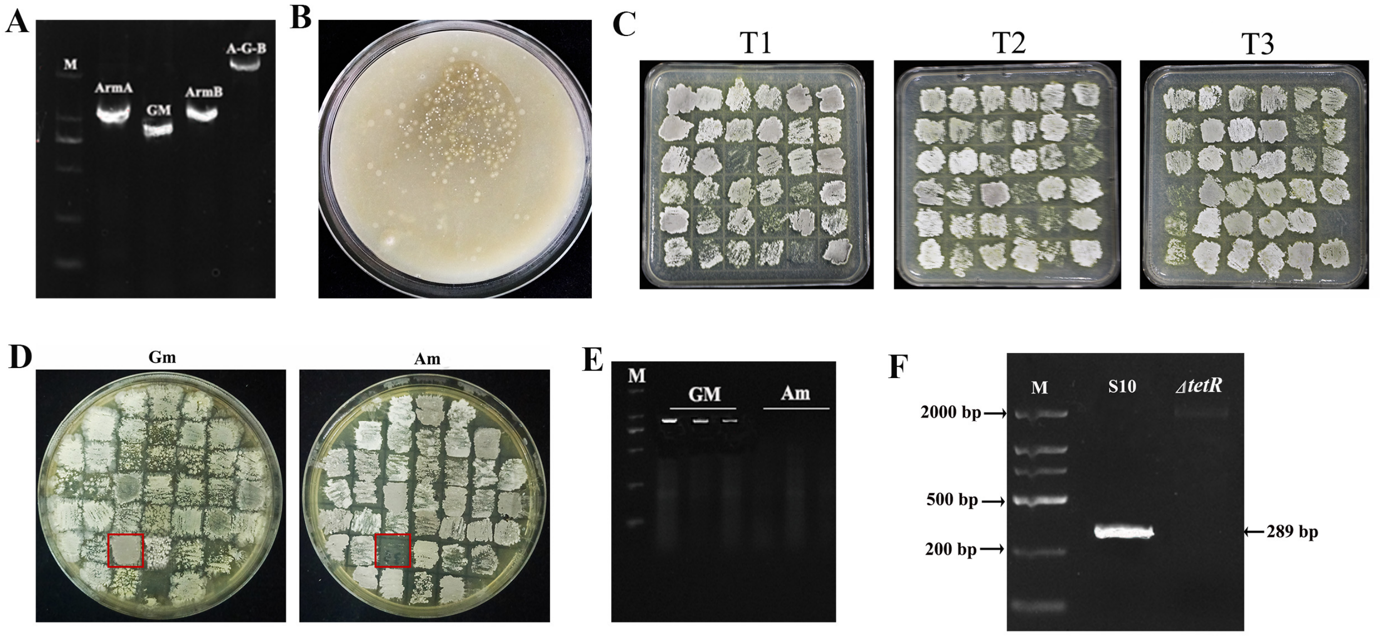

Figure S1. The mutant Δ*tetR* was constructed. (A) The PCR production of ArmA, GM, ArmB, and recombinant plasmid pKC1139-AGB. (B) Transconjugant. (C) The generation of transconjugants. (D) Single spore streaked on medium containing Am and Gm, respectively. (E, F) PCR validation of mutant strain.

Reference

1. Chen, J.; Lan, X.J.; Jia, R.M.; Hu, L.F.; Wang, Y. Response surface methodology (RSM) mediated optimization of medium components for mycelial growth and metabolites production of *Streptomyces alfalfae* XN-04. *Microorganisms* **2022**, *10*, 1854. https://doi.org/10.3390/microorganisms10091854.
